# Effects of exercise habituation and aging on the intersegmental coordination of lower limbs during walking with sinusoidal speed change

**DOI:** 10.1186/s40101-022-00298-w

**Published:** 2022-06-08

**Authors:** Daijiro Abe, Kiyotaka Motoyama, Takehiro Tashiro, Akira Saito, Masahiro Horiuchi

**Affiliations:** 1 Center for Health and Sports Science, Kyushu Sangyo University, 2-3-1 Matsukadai, Higashi-ku, Fukuoka, 813-8503 Japan; 2 Human Robotics Research Center, Kyushu Sangyo University, Fukuoka, Japan; 3 Division of Human Environmental Science, Mount Fuji Research Institute, Fuji-yoshida, Yamanashi Japan

**Keywords:** Whole-body coordination, Interlimb coordination, Lack of exercise, Erect bipedalism, Locomotion, Biomechanics, Dynamic balance

## Abstract

**Background:**

The time courses of the joint elevation angles of the thigh, shank, and foot in one stride during walking can be well approximated by a “*plane*” in a triaxial space. This intersegmental coordination (IC) of the lower limb elevation angles is referred to as the planar covariation law. We examined the effects of exercise habituation and aging on the thickness of the IC *plane* of the lower limbs under sinusoidal speed changing conditions.

**Methods:**

Seventeen sedentary young (SY), 16 active young (AY), and 16 active elderly (AE) adults walked on a treadmill in accordance with a sinusoidal speed changing protocol at 120, 60, and 30 s periods with an amplitude of ± 0.56 m·s^−1^. Motion of the lower limbs from the sagittal direction was recorded to calculate the elevation angles of the lower limbs. When the best-fit IC *plane* was determined, the smallest standard deviation of the IC *plane* was considered as the anteroposterior gait variability of the lower limbs. The coefficient of variance of the step width was also quantified to evaluate the lateral step variability (CV_SW_).

**Results:**

The standard deviation of the IC *plane* was significantly greater in the order of SY, AY, and AE, regardless of the sinusoidal wave periods of the changing speed. The CV_SW_ was not significantly different among the three groups.

**Conclusions:**

Exercise habituation influences anteroposterior gait variability of the lower limbs, but not lateral step variability, even in young adults. Given these, gait adaptability for sinusoidal speed changes does not always decline with aging.

**Trial registration:**

UMIN000031456 (R000035911; registered February 23, 2018).

**Supplementary Information:**

The online version contains supplementary material available at 10.1186/s40101-022-00298-w.

## Background

Erect bipedalism is an intrinsic human gait pattern. One of the biological benefits of the erect bipedalism has been reported to be more economical than quadrupedalism [1]. However, it also underlies several functional disadvantages, such as knee-back pain [2], slower top speed compared with other mammals [3–5], and increased fall risk [6]. In human gait, the time courses of the elevation angles of the thigh, shank, and foot in one gait cycle can be well approximated by a *plane* in a triaxial space [7], which is referred to as the planar covariation law (PCL) [8–11]. This spatiotemporal intersegmental coordination (IC) of the elevation angles of the lower limbs functions as the whole-body coordination, and it contributes to the simplification of the control of locomotion. The spatiotemporal IC was disrupted when one leg was perturbed [9, 10]. Thus, maintaining the *plane* planarity of the IC during the entire gait cycle could represent each individual’s gait stability. It has been reported that a rehabilitation program in conjunction with a habitual exercise improved gait stability in the elderly [12], middle-aged [13], and young patients with chronic ankle instability [14, 15] or cerebral palsy [16]. Postural balance was less stable in untrained elderly adults than in trained elderly and young adults [17, 18]. Recreational soccer training in sedentary young men improved postural balance [19]. These previous findings suggest that an exercise habituation could be associated with gait stability even in healthy young adults who do not suffer from any chronic disability. However, it is noteworthy that these previous studies used different indices and tested speeds in each study [12–19], indicating that it is impossible to make a direct comparison of those data. One of the benefits of the PCL is that it can be established regardless of gait speed [11].

To date, aging has been acknowledged as the major cause of gait instability in healthy adults (e.g., [20]). However, an age-related effect on the ability to walk begin to decline, at least, around the mid-forties [21]. Given these backgrounds, it is currently debated on whether the PCL concept can detect gait variability differences between young and elderly adults [9, 22–25]. Some of these previous studies found an age-related difference of the *plane* planarity [22, 23], but others did not [9, 24, 25]. These discrepancies indicated that factors other than aging may influence gait variability. We also questioned whether the IC thickness represents the whole-body gait variability. In this sense, step width (SW) variability should also be evaluated because it would reflect lateral gait variability which is associated with fall risks [20, 26–28]. Another important point is that age-related variability of the major gait parameters are likely to be greater at faster compared with slower speeds [29–31]. This could be attributed to the fact that the neuromuscular system does not have enough time to accomplish appropriate adjustment of the lower limbs at faster speeds. However, little information has been available about the effects of changing speed on the gait stability [9, 10]. These studies especially investigated a perturbation-induced compensatory strategy during walking when one leg was perturbed, thus indicating that the effects of gradual speed changes on the *plane* planarity during walking in the absence of perturbation have not yet been investigated. Herein, a sinusoidal speed-changing protocol will be beneficial because it involves both slower and faster speeds. It also requires an adaptability for a consecutive intersegmental limb adjustment during walking. Therefore, the purpose of this study was to test a hypothesis that anteroposterior and/or lateral gait variability would be greater in the sedentary young (SY) than in the active young (AY) adults in conditions where gait speed was sinusoidally changed. We also hypothesized that anteroposterior and/or lateral gait variability would be greater in the active elderly (AE) adults compared with other young groups.

## Methods

### Participants

This study involved 49 participants, grouped by age and exercise habituation into 17 SY (eight males and nine females; 20.3 ± 0.9 years old, mean ± standard deviation [SD]), 16 AY (ten males and five females, 19.9 ± 0.6 years old), and 16 AE participants (10 males and six females, 74.1 ± 4.6 years old). The ethical committee established in Kyushu Sangyo University approved all procedures (no. 2019-0002). In adherence to the Declaration of Helsinki, all participants provided written informed consent after being provided with information about the purposes, experimental protocols, and possible risks of this study.

The SY participants had no current experience in habitual dynamic exercise except for that occurred during their participations in physical education classes during their schooldays, indicating that the SY participants were far from the minimum recommendation of the WHO guideline (at least 150 min of weekly moderate-intensity aerobic physical activity [32]). The AY participants belonged to competitive sports teams and were associated with dynamic exercises (e.g., volleyball, soccer, tennis, and track and field) in their high school days. They are still regularly engaging in sports activities at a recreational level (90–120 min a day, 2–4 times a week) after university admission. The AE participants belong to a community walking and mountain climbing club in their local community (brisk walking habituation for 40–60 min a day, 5–7 days a week). The enrollment criteria in this club required that the person could climb up the mountains located around the city (300–500 m above the sea level) without an assistive device and other aid. Therefore, the AY and AE participants apparently exceeded WHO’s physical activity recommendation [32].

### Protocols and motion analysis

**Fig. 1:**
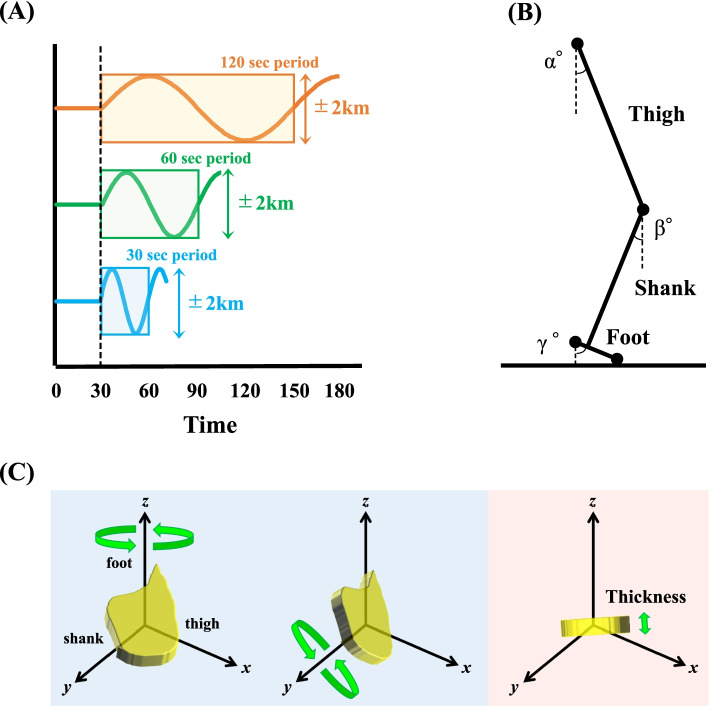
Schematic illustrations of the setup and data analysis. **A** Sinusoidal speed changes at different periods (120, 60, and 30 s). *Shaded areas* are the analyzed sections. **B** Definitions of elevation angles at the thigh, shank, and foot. **C** The calculation procedure of the best-fit loop of the elevation angles of thigh, shank, and foot is plotted in a squared 3D space as a “*plane*” (shaded in yellow). This *plane* was simultaneously rotated around the *z* and *y* axes (shaded in blue). The *z* angle, at which the smallest standard deviation was obtained, is regarded as the “thickness” of the spatiotemporal intersegmental coordination (shaded in red)

The participants wore a half spats, compression shirts, and the same shoes (but different sizes) (ADIZERO-RC, Adidas, Herzogenaurach, Germany). The use of the PCL concept was effective to clarify the adaptability of continuous speed-changing tasks because an abrupt change in the gait speed is likely to violate perturbed leg’s IC [9, 10]. After warming-up and habituation on a motor-driven treadmill (TKK3080, Takei Scientific Instruments, Niigata, Japan), the preferred walking speed (PWS) of each individual was determined [33] for the baseline speed (i.e., midpoint speed during sinusoidal walking). A week later, the participants freely walked on the same treadmill at the baseline speed for 30 s, and then the treadmill speed was changed in a sinusoidal manner of 120, 60, and 30 s periods with amplitudes equal to ± 0.56 m·s^−1^ (± 2 km·h^−1^) in a randomized order (Fig. 1A).

Twelve reflective markers were placed on both shoulders (acromion), lateral greater trochanters, knees (lateral femur epicondyle), ankles (lateral malleolus), toes (toe of each shoe), and heels (backend of each shoe) [8, 11]. Four additional markers were placed on each corner of the treadmill. Motion data were captured with eight high speed cameras (Kestrel300, MAC3D System, Rohnert Park, CA, USA) with a sampling rate of 100 Hz. The root mean square errors in the calculation of the three-dimensional (3D) marker locations were found to be < 1.0 mm. The entire gait cycle, which was defined from the heel-contact to the toe-off, was divided distinct parts in the range of 0–100%. The 3 × 3 matrix of the elevation angles of the right thigh, shank, and foot (30-120 s) was computed from the marker locations (Fig. 1B) at every time frame. The best-fit 3D covariation loop did not perfectly lie on a *plane* [7–11]. Additionally, the *plane* seems to fluctuate during walking according to a sinusoidal speed-changing manner (Fig. S1). Considerably large variations in the IC “thickness” were observed when the shoe sole slightly rubbed the treadmill belt before the heel strike. To avoid these incomplete motions, each sinusoidal cycle was repeated continuously four times. Although the first sinusoidal period was fundamentally used for subsequent analyses, the second, third, or fourth cycle was used only when perturbations occurred in the earlier cycles. Thus, we did not use the largest standard deviation or mean value to represent the IC thickness, and the smallest standard deviation of the fluctuating *plane* in one gait cycle was regarded as the IC thickness (Fig. 1C).

In a practical computational calculation, the best-fit 3D approximation of the angular covariation is not a dimensionless *plane*. Thus, according to the definition of Euler’s angle, when the best-fit *plane* of the 3D covariation was detected, it was rotated around the *z*-axis (foot elevation angle) as follows,

1 $$\left(\begin{array}{c}\alpha z\\ {}\beta z\\ {}\gamma z\end{array}\right)=\left(\begin{array}{ccc}\cos \theta z& \sin \theta z& 0\\ {}-\sin \theta z& \cos \theta z& 0\\ {}0& 0& 1\end{array}\right)\left(\begin{array}{c}\alpha \\ {}\beta \\ {}\gamma \end{array}\right)$$

where *α*, *β*, and *γ* are the original best-fit covariations, and *α*_*z*_, *β*_*z*_, and *γ*_*z*_ are the covariations after rotated around the *z*-axis. The matrix described by Eq. (1) was simultaneously rotated around the *y*-axis (knee elevation angle) as follows,

2 $$\begin{pmatrix}\alpha y\\\beta y\\\gamma y\end{pmatrix}=\begin{pmatrix}\cos\theta y&0&\sin\theta y\\0&1&0\\-\sin\theta y&0&\cos\theta y\end{pmatrix}\begin{pmatrix}\alpha z\\\beta z\\\gamma z\end{pmatrix}$$

where *α*_*y*_, *β*_*y*_, and *γ*_*y*_ are the covariations after rotation around the *y*-axis. Thus, the *plane* was rotated by a combination of the matrices *1* and *2* as follows:

3 $$\left(\begin{array}{c}{\alpha}^{\prime}\\ {}{\beta}^{\prime}\\ {}{\gamma}^{\prime}\end{array}\right)=\left(\begin{array}{ccc}\cos \theta y& 0& \sin \theta y\\ {}0& 1& 0\\ {}-\sin \theta y& 0& \cos \theta y\end{array}\right)\left(\begin{array}{ccc}\cos \theta z& \sin \theta z& 0\\ {}-\sin \theta z& \cos \theta z& 0\\ {}0& 0& 1\end{array}\right)\left(\begin{array}{c}\alpha \\ {}\beta \\ {}\gamma \end{array}\right)=\left(\begin{array}{ccc}\cos \theta y\ \cos \theta z& \cos \theta y\ \sin \theta z& \sin \theta y\\ {}-\sin \theta z& \cos \theta z& 0\\ {}-\sin \theta y\ \cos \theta z& -\sin \theta y\ \sin \theta z& \cos \theta y\end{array}\right)\left(\begin{array}{c}\alpha \\ {}\beta \\ {}\gamma \end{array}\right)$$

Given that both rotation angles, “*θ*_*z*_” and ”*θ*_*y*_,” ranged from 0° to 179°, 32400 (180 × 180) combinations can be defined. The IC thickness was then calculated in a non-arbitrary computational space.

The SW was quantified as the lateral distance between both heel makers in each step [20], and it was less dependent on the gait speed [28]. Thus, the SW was measured during the whole first period (30–120 s), and the coefficient of variance of the SW (CV_SW_; %) and “actual length” of the SW variability itself (cm) were then calculated.

### Statistical analysis

The software G*Power 3.1 [34] was used to estimate the required number of participants with an effect size of 0.25 [35], an alpha level of 0.05, and a statistical power of 80%; at least 12 participants would be needed in each group. Two-way repeated measures analysis of variance (ANOVA) within periods and between groups was performed on the dependent variables using ANOVA 4 on the web. Eta-squared values (*η*^2^) were also presented [35]. If a significant *F* value was obtained on the dependent variables, Ryan’s post hoc test was applied to the appropriate datasets to detect significant mean differences. Its statistical power has been reported to be equivalent to Tukey’s post hoc test [36] and can be used regardless of the data distribution [36]. That is, assessment of data normality is not always required if Ryan’s post hoc test is applied for the data sets. Cohen’s *d* values were further provided for the post hoc test [35]. Statistical significance was set at *p* < 0.05. All data were presented as mean ± SD.

## Results

**Fig. 2:**
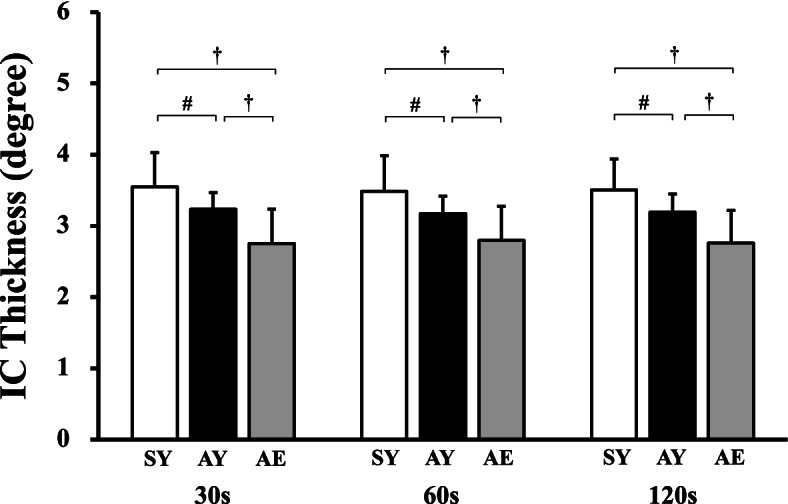
Comparisons of the thickness values associated with spatiotemporal intersegmental coordination (IC). Participants walked according to a sinusoidal speed-changing protocol for time periods of 30 s, 60 s, and 120 s period, respectively. SY, sedentary young (*white bars*); AY, active young (*black bars*); and AE, active elderly adults (*gray bars*), respectively. ^#^*p* < 0.05 and ^†^*p* < 0.01, respectively. Values are means ± standard deviations

**Fig. 3:**
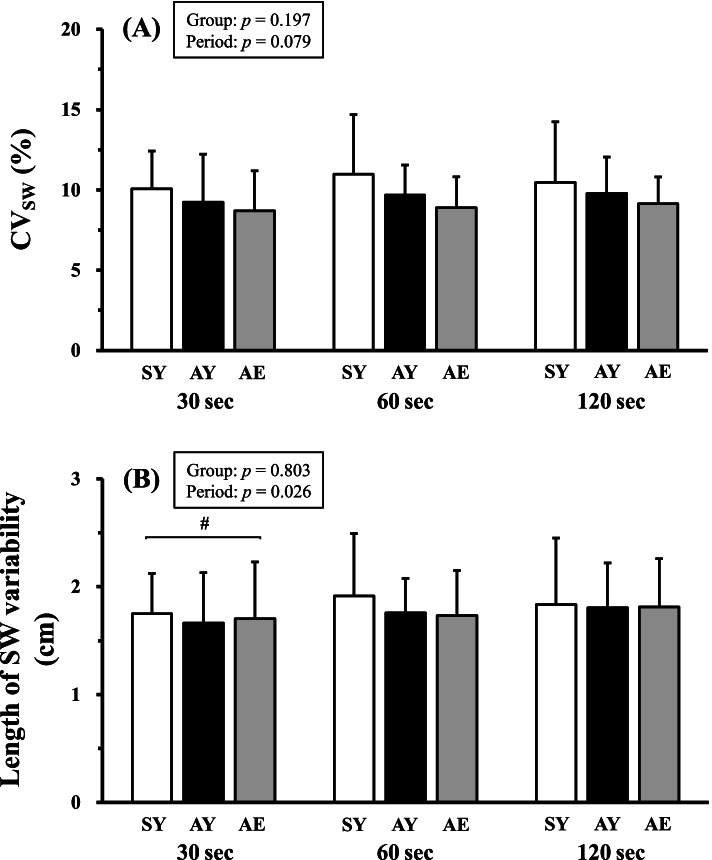
Comparisons of step width (SW) variability. Coefficient of variance values of the SW (CV_SW_; %) and actual length of the SW variability (cm) were compared between groups and periods. **A** There were no significant differences in the CV_SW_ among sedentary young adults (SY, *white bars*), active young adults (AY, *black bars*), and active elderly adults (AE, *gray bars*). **B** The length of the SW variability was not significantly different between groups, but it was significantly smaller in the 30 s period compared with the other periods. ^#^ indicates *p* < 0.05. Values are means ± standard deviations

The baseline speed was 1.37 ± 0.06, 1.30 ± 0.07, and 1.08 ± 0.07 m·s^−1^ in the AY, SY, and AE adults, respectively. As shown in Fig. S1, the IC *plane* continuously lurched during walking under sinusoidal speed-changing conditions. A significant main effect of participant groups was observed in the thickness of the IC *plane* (*F*_2,46_ = 13.917, *p* < 0.001, *η*^2^ = 0.367; Fig. 2), but no significant interactions (*F*_4,92_ = 1.714, *p* = 0.154, *η*^2^ = 0.002; Fig. 2) and main effects of sinusoidal walking periods were observed (*F*_2,46_ = 1.258, *p* = 0.289, *η*^2^ = 0.001; Fig. 2). A post hoc test revealed that the thickness of the IC *plane* was significantly greater in the SY than the AY (*t*_97_ = 2.227, *p* = 0.031, *d* = 0.851; Fig. 2) and AE (*t*_97_ = 5.281, *p* < 0.001, *d* = 1.608; Fig. 2). It was also greater in the AY than in the AE (*t*_97_ = 3.009, *p* = 0.004, *d* = 1.166; Fig. 2). The CV_SW_ was not significantly different between periods (*F*_2,46_ = 2.607, *p* = 0.079, *η*^2^ = 0.059; Fig. 3A) and groups (*F*_2,46_ = 1.682, *p* = 0.197, *η*^2^ = 0.007; Fig. 3A). The actual length of the SW variability was not significantly different between groups (*F*_2,46_ = 0.221, *p* = 0.803, *η*^2^ = 0.008; Fig. 3B), but it was significantly smaller in the 30 s period compared with the other periods (*F*_2,46_ = 3.785, *p* = 0.026, *η*^2^ = 0.011; Fig. 3B). A post hoc test revealed that the actual length of the SW variability was significantly smaller in the 30 s period than in the 60 s period (*t*_96_ = 2.209, *p* = 0.030, *d* = 0.193; Fig. 3B) and 120 s period (*t*_96_ = 2.526, *p* = 0.013, *d* = 0.170; Fig. 3B). These *F* and *p* values of 2-way ANOVA were summarized in Table S1.

## Discussion

In support of our first hypothesis, the IC thickness was significantly greater in the SY compared with the AY during the application of the sinusoidal speed-changing protocol at any measured period (Fig. 2). This finding suggests that the anteroposterior gait variability was not influenced by aging alone. The ability to walk began to decline at around the mid-40s [21]. Many previous studies which investigated gait stability aimed to avoid falling and/or reducing fall risks in the elderly adults [8–15, 20, 22–31]. By contrast, it has been reported that middle-aged [6, 37] and young [6, 37, 38] adults also fall in their daily lives. Fall accidents in young adults were more frequent as physical activity levels increased [38]. These incidents tended to occur mostly due to perturbed surface conditions [6]. Thus, little is known about the effects of exercise habituation on gait variability in healthy young adults without any chronic gait disability. Physiological variability is sometimes associated with adaptability or flexibility (e.g., heart rate variability (HRV)) [39–41]. In the HRV literatures, a greater variability magnitude means greater adaptability for exercise tolerance [39]. However, some considerations are necessary with regard to gait variability because excessive gait variability is likely linked with an increase in fall risk [26, 27], which is one of the major biological disadvantages of erect bipedal locomotion. Owing to a limited treadmill capacity, all previous studies using the PCL concept have employed a constant speed protocol [7–11, 22–25, 28, 30]. Conversely, we focused on the lower limb coordination variability when the walking speed was sinusoidally changed at different three periods. Thus, gait speed was gradually and continuously changed in our study, indicating that our protocol did not emulate exactly unexpected slip-like perturbation [9, 10]. Our present results demonstrate that exercise habituation affected anteroposterior gait variability of the lower limbs even in the young generation (Fig. 2), which is supported by the results of a previous study [19]. This functional difference between SY and AY was not influenced by sinusoidally speed-changing periods which ranged from 30 to 120 s (Fig. 2). These results may indicate that anteroposterior gait adaptability of the lower limbs is independent of the rate of change of speed in young adults.

Although a trend for a greater CV_SW_ value was observed in the order of SY, AY, and AE (*p* = 0.079; Fig. 3A), the CV_SW_ values were not significantly different between the groups. A recent meta-analysis study reported that the “optimal reference range of the SW variability” was within 1.97 cm in young adults [20]. In our present study, the actual length of the SW variability was not significantly different between the groups (Fig. 3B), and it was ≤ 1.97 cm in all measured periods (Fig. 3B). Given these results, lateral gait variability was not influenced by exercise habituation in the case of the sinusoidal speed-changing protocol.

To our surprise, the *plane* planarity (IC thickness) evaluated by the PCL concept was significantly smaller in the AE than other young groups regardless of the sinusoidal periods (Fig. 2), indicating that our second hypothesis was rejected. These results seemed to indicate that the AE adults presented greater anteroposterior gait variability compared with young adults. However, careful considerations are necessary because the AE adults are likely to stabilize their ankle joints by means of a coactivation of the *soleus* and *tibialis anterior* [42, 43], thus potentially resulting in a narrower range of the ankle joint motion. In fact, there was a smaller time delay between thigh and shank motions in the elderly adults than young adults at any gait speed [22]. Such a greater time delay in the shank-foot coordination provided greater distortion of the planarity of the IC *plane* [22]. Thus, age-related increase in a stabilization of the ankle joint during walking could be a strategy to avoid falling in the elderly adults. Given that the narrower range of the ankle joint elevation angle can easily synchronize other joint (knee and hip joints) elevation angles [8, 20], it is perceivable that the AE adults were associated with a smaller anteroposterior gait variability compared with other young groups (Fig. 2). In addition, the AE adults walked on the treadmill at several sinusoidal speed-changing conditions at a CV_SW_ of ~ 9% (Fig. 3A). These values were quite smaller than those of previous studies even when walking at constant speeds [23, 26, 44, 45], and the actual length of the SW variability was small enough with respect to the optimal reference range of the SW variability (2.50 cm) in the case of elderly adults [20]. It has already been well acknowledged that lateral gait stabilization can be established by an interaction between active control of the central nervous system and passive musculoskeletal dynamics [46]. A decrease in the sensorimotor precision potentially results in greater CV_SW_ values [47]. Indeed, the age difference did not always explain the CV_SW_ between young and elderly adults [31], which was in good agreement with our present results (Fig. 3A). Based on these theoretical and experimental backgrounds, lateral gait variability in our AE adults was well maintained despite of aging.

Methodological considerations should not be neglected. Some previous studies have presented that the elderly adults exhibited different *plane* planarities compared with younger adults [22, 23]. Such an age difference could be derived from reduced ankle torque in elderly adults [48], resulting in a gait instability especially at faster speeds [26–31]. In contrast, some others did not observe any age differences in gait variability which was evaluated based on the *plane* planarity [9, 24, 25], despite the fact that these studies employed a similar technique to that employed herein. This discrepancy suggests that gait variability may depend on the protocol (task) itself. A novelty of the present study refers to the use of a sinusoidal speed changing protocol with different wave periods because it consecutively required spatiotemporal interlimb adjustment. Thus, the sinusoidal speed-changing protocol used in the present study may be more sensitive in its ability to detect IC thickness difference rather than a use of constant speed protocol that was previously employed [7–11, 22–25]. In other words, greater gait adaptability observed in active adults for sinusoidal speed changes may function as one of the functional potentialities [49] to avoid falling regardless of aging. In addition, no significant differences in the IC thickness were found among the measured three wave periods in each group (Fig. 2), suggesting that the ability for spatiotemporal gait adjustment is inherent in each individual over 30 s period under the sinusoidal speed-changing protocol. As a limitation of this study, an amplitude of the sinusoidal speed change was set at ±0.56 m·s^−1^ in all measured periods in the present study. This was set to avoid gait transition, but effects of the amplitudes of the sinusoidal speed changes should be further investigated in future studies.

## Conclusions

The IC thickness values were significantly greater in the order of SY, AY, and AE, regardless of sinusoidal wave periods. The CV_SW_ values were not significantly different among the three studies groups. These results suggest that anteroposterior gait variability, but not lateral gait variability, was influenced by exercise habituation even in young adults without any chronic disability when walking under sinusoidal speed-changing protocol. Given these, individual gait adaptability does not always decline with aging.

## Supplementary Information

**Additional file 1: Table S1.** Summary of statistical results.

**Additional file 2: Figure S1.** Fluctuating intersegmental coordination of the lower limbs during walking with sinusoidal speed-changing protocol. Left, middle, and right panels present representative participants in sedentary young (SY), active young (AY), and active elderly (AE) groups, respectively. Red, purple, and green loops refer to the 30 s, 60 s, and 120 s periods, respectively.

## Data Availability

The data of this study are available from the corresponding author upon reasonable request.

## Abbreviations

ANOVA: Analysis of variance
AE: Active elderly
AY: Active young
CV_SW_: Coefficient of variance of step width
HRV: Heart rate variability
IC: Intersegmental coordination
PCL: Planar covariation law
PWS: Preferred walking speed
SD: Standard deviation
SW: Step width
SY: Sedentary young
3D: Three-dimensional
