# Supplementary material for: Effects of exercise habituation and aging on the intersegmental coordination of lower limbs during walking with sinusoidal speed change
Source: J Physiol Anthropol. 2022 Jun 8;41:24. doi: 10.1186/s40101-022-00298-w (PMC9175341; doi:10.1186/s40101-022-00298-w)
Supplement: Supplementary file 1 — Additional file 1: Table S1. Summary of statistical results. [file 40101_2022_298_MOESM1_ESM.docx]

| **Table S1** Summary of statistical results. | |  |  |
| --- | --- | --- | --- |
|  | Group (SY/AY/AE) | Period (30/60/120 s) | Group × Period interaction |
|  | *F*-value (*p*-value) | *F*-value (*p*-value) | *F*-value (*p*-value) |
| IC thickness | **13.917 (< 0.001)** | 1.258 (0.289) | 1.714 (0.154) |
| CV_SW_ | 1.682 (0.197) | 2.607 (0.079) | 0.580 (0.678) |
| Length of SW variability | 0.221 (0.803) | **3.785 (0.026)** | 0.723 (0.579) |
| IC, intersegmental coordination; CV_SW_, coefficient of variance of the step width, respectively. | | | |
